# Work outside of work: after-hours use of the electronic medical record among residents at an academic veterinary teaching hospital

**DOI:** 10.3389/fvets.2026.1951548

**Published:** 2026-09-22

**Authors:** Sanchit Shah, Kim Love, Donnie Jenkins, Katherine E. McCool

**Affiliations:** 1 Department of Computer Science, College of Engineering, North Carolina State University, Raleigh, NC, United States; 2 Consultant, Athens, GA, United States; 3 NC State Veterinary Hospital, College of Veterinary Medicine, North Carolina State University, Raleigh, NC, United States; 4 Department of Clinical Sciences, College of Veterinary Medicine, North Carolina State University, Raleigh, NC, United States

**Keywords:** clinical workload, documentation, electronic medical record, house officer, house officer wellness, veterinary teaching hospital

## Abstract

The electronic medical record (EMR) is a major contributor to clinician workload, with substantial documentation often occurring outside scheduled patient care hours. In human medicine, after-hours EMR use has been associated with clinician burnout, yet it has not been objectively quantified among veterinary residents. The purpose of this study was to quantify after-hours EMR use among veterinary residents, to compare this across specialties and years of training, and to establish baseline metrics for future studies evaluating documentation efficiency and workflow interventions. EMR login metadata were extracted for 39 residents representing seven specialties at a single academic veterinary teaching hospital between July 2023 and July 2024. Rule-based algorithms quantified weekday after-hours use (pajama time; PJT) and weekend time (WT; weekend EMR use). Repeated-measures hurdle models were used to evaluate associations between specialty, residency year, and after-hours EMR use. Specialty was significantly associated with both the likelihood and duration of after-hours EMR use (*p* < 0.001). Radiology residents consistently demonstrated the lowest after-hours EMR use, whereas residents in surgery, cardiology, neurology, and small animal internal medicine accumulated substantially greater after-hours EMR time. Among residents who used the EMR during after-hours time, median weekday PJT was 13.57 h/month and median weekend EMR use was 23.38 h/month. First-year residents were more likely than third-year residents to access the EMR during weekday PJT (OR 4.57, 95% CI 1.69–12.36; *p* = 0.002), although the duration of after-hours EMR use did not differ by residency year. Veterinary residents spend substantial time using the EMR outside scheduled working hours, with marked variation across specialties. Objective EMR metadata provide a scalable, objective approach for quantifying documentation workload and establishes a baseline for evaluating interventions aimed at improving workflow efficiency and documentation practices within veterinary training programs.

## 1 Introduction

The electronic medical record (EMR) is an important component of clinician workload, and time spent on the EMR constitutes a substantial proportion of a clinician’s working hours (1). Recent estimates have shown that physicians spend over half of their workday on the EMR (2) and this number continues to grow (3, 4). Time spent on the EMR may include a variety of tasks such as patient notes, case preparation, prescribing medications, and responding to inbox messages, and is influenced by multiple factors, including specialty (5, 6), gender of the provider (7), and system-level factors (8). One major advantage of EMR over traditional paper records is the increased portability which allows clinicians to complete their work outside the physical walls of facilities. While this can lead to greater flexibility in some circumstances, this may also cause an expansion into time outside of scheduled shift time, leading to an increase in “work outside of work” (9). One recent study showed that almost half of physicians’ EMR use occurred outside of scheduled patient hours (2). Various metrics have been used to describe this “work outside of work” including: pajama time (10) which refers to (work in the evenings during the week), and weekend time (work during unscheduled weekends), and Clinician-Logged-in Outside Clinic (11).

Veterinary medicine has seen a similar rise in EMR adoption (12), and EMRs are now common in veterinary teaching hospitals, where veterinary residents are trained and maintaining timely and accurate medical records are a key component of residency training programs (13). Client access to providers has also increased, with rising messages to providers via the medical record (14) and increased amount of time spent triaging inbox messages (15). Despite these parallels to human medicine, no studies to date have examined the pattern of “work outside of work” among veterinary residents. The purpose of this study was to quantify after-hours EMR use among veterinary residents, to compare this across specialties and years of training, and to establish baseline metrics for future studies evaluating documentation efficiency and workflow interventions.

To do this, we looked at 2 metrics of describing after-hours work: pajama time (PJT), and weekend time (WT).

## 2 Materials and methods

This study was conducted at the College of Veterinary Medicine at North Carolina State University and received exemption from IRB approval (# 28126). Participants from study included residents from the following specialties: small animal internal medicine (SAIM), radiology, small animal surgery (SAS), anesthesiology, cardiology, medical oncology, and neurology. This study took place during the time period of July 1 2023–July 1 2024.

### 2.1 EzyVet user data extraction

SAP Crystal Reports 2016 (SAP Corporation, Newtown, PA, USA) was used to capture and export EMR user metadata from EzyVet (IDEXX, Frisco, TX, USA) for the desired participants and date ranges using structured query language code for the desired participants and date ranges. The exported data contained information about each selected users’ “login time” and “last active” time. Using a hospital employee roster, the corresponding author (KEM) identified each trainee by specialty and training year. The dataset was filtered to remove missing or incomplete values, and the data were de-identified for statistical analysis.

### 2.2 Calculation of EMR hours outside of work

Pajama time (PJT).

In this study, PJT was defined as the time spent by an individual resident on the EMR before 7:00 a.m. and after 7:00 p.m. on weekdays (Monday–Friday). An algorithm was developed using the R statistical computing language (version 4.5.1) (16) to calculate the amount of PJT for each resident, and PJT was defined as the amount of time in minutes that an individual spent logged into the EMR outside of these specified hours. Session-level PJT was calculated using a rule-based algorithm. If both login and logout times occurred before 7:00 a.m., the total session duration was included. If both login and logout times occurred after 7:00 p.m., the total session duration was included. If a session started before 7:00 a.m. and ended between 7:00 a.m. and 7:00 p.m., PJT was calculated as the interval from login time to 7:00 a.m. If a session started between 7:00 a.m. and 7:00 p.m. and ended after 7:00 p.m., PJT was calculated as the interval from 7:00 p.m. to logout time. Sessions occurring entirely between 7:00 a.m. and 7:00 p.m. were excluded from calculation.

Weekend Time (WT).

Weekend time was defined as any EMR usage occurring between 12:00 a.m. Saturday and 11:59 p.m. Sunday, and an algorithm was developed using similar methods as described above for WT. For sessions occurring on weekends, the full session duration was included regardless of time of day.

### 2.3 Data preprocessing and outlier removal

Following calculation of both PJT and WT at the session level, some overlapping sessions were identified due to users logging in on multiple devices at the same time. To address this, for any set of overlapping sessions, that set of sessions were removed from the data and replaced with one session duration with the earliest login time and the latest logout time from the set of overlapping sessions.

Distributions of both pajama time and weekend time were also visualized using R. Following calculation of both PJT and WT at the session level (each observation representing a single session), distributions of both variables were visualized. Due to the temporal nature of session duration data, both variables had substantial right skew. A log-transformed outlier detection and removal method was used to clean the data. Sessions were log-transformed and the distributions for PJT and WT were each visualized. Outliers were defined here as observations that were clearly visually separated from the continuous distribution, using a standard threshold of values 1.5 times the interquartile range above the 75th percentile of the log transformed data. These points were removed from the dataset prior to further analysis. Session-level data were aggregated to the resident-month level by summing each session for PJT and WT for each resident within each calendar month.

### 2.4 Statistical analysis

Due to the relatively large percentage of 0 s (more than 10% of both PJT and WT), a two-stage double hurdle model was used to characterize the outcome distribution. The probability of any EMR use (non-zero vs. zero) for PJT and separately for WT was analyzed using a binary logistic generalized estimating equation (GEE). This analysis estimated the probability that an individual spent any PJT or WT in the EMR based on their year and specialty. Among observations with non-zero EMR use, time spent in the EMR was analyzed using marginal models with repeated measures to incorporate correlation within individuals over time. Monthly EMR time was natural log-transformed to address right-skewness, and model estimates were back-transformed and are presented as model-estimated medians with 95% confidence intervals (Figure 1).

**Figure 1:**
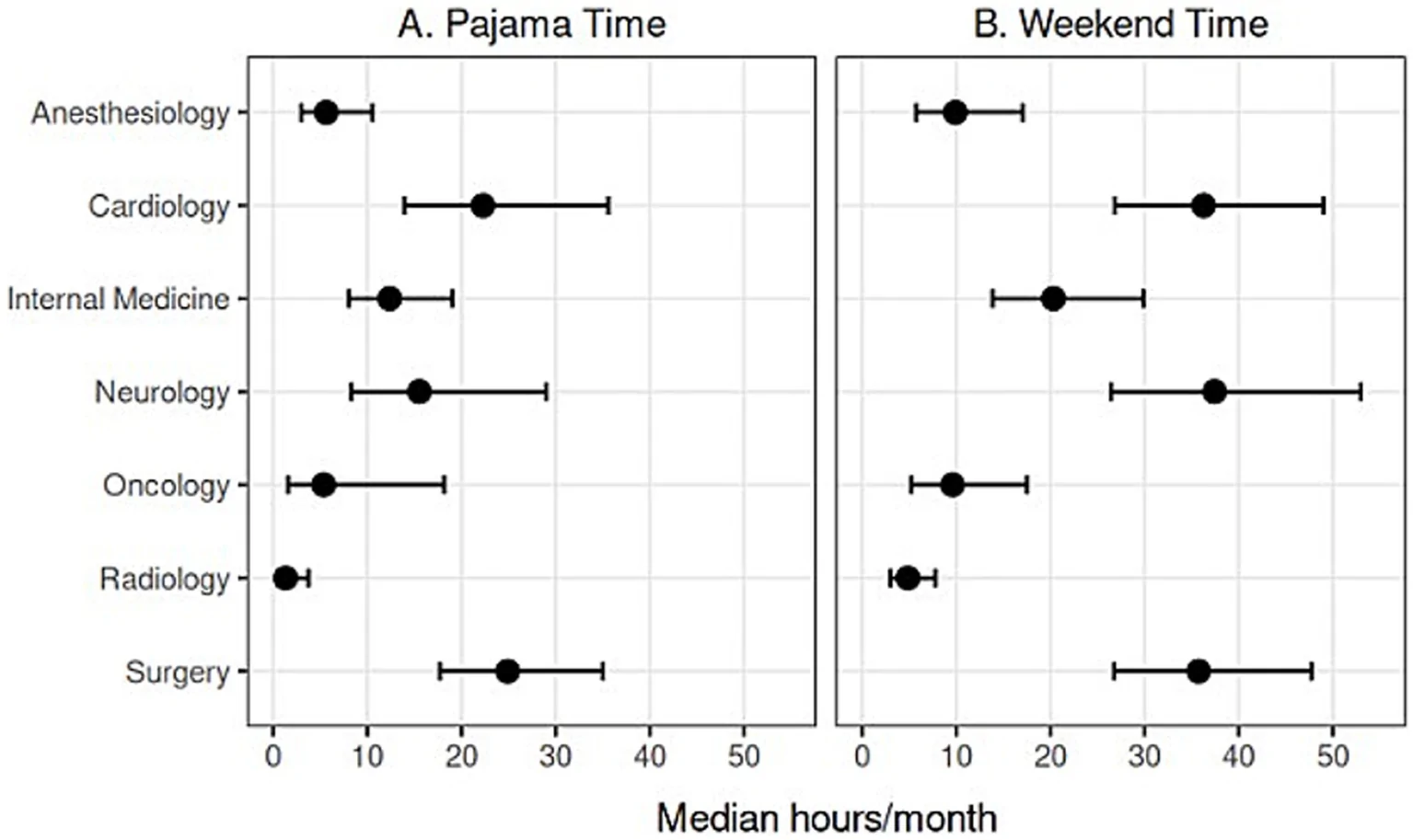
Model-estimated median EMR use outside work hours by specialty (*n* = 39 residents). Error bars signify 95% confidence intervals.

For all models, residency year and specialty were included as main effects; interaction terms were not included due to small sample sizes within some specialty and year combinations. Correlation structures were selected based on likelihood-based fit criteria, using the quasi-information criterion (QIC) for binary models and the Akaike information criterion (AIC) for continuous models. An autoregressive (AR1) correlation structure was selected for binary outcomes, and a compound symmetry structure was selected for continuous outcomes. For binary models where certain specialties had 100% EMR usage, a single observation was randomly reassigned as a zero to allow for model convergence; this approach introduces minimal bias given the total sample size. Heterogeneous variance structures were incorporated where appropriate to account for different variability in total hours within different specialties. All analyses were performed in R (version 4.5.3) using the nlme (17) and geepack (18) packages. Statistical significance was set at *p* < 0.05.

## 3 Results

### 3.1 Participants and specialties

A total of 39 residents from 7 specialties were enrolled in the study. These included: SAIM (*n* = 10), radiology (*n* = 7), SAS (*n* = 6), anesthesiology (*n* = 5), cardiology (*n* = 4), medical oncology (*n* = 4), and neurology (*n* = 3). Table 1 provides a complete breakdown of residents by specialty and year.

**Table 1:** Distribution of residents by specialty and year.

Specialty	Year 1	Year 2	Year 3	Total
Anesthesiology	1	2	2	5
Cardiology	2	1	1	4
SAIM	4	3	3	10
Neurology	1	1	1	3
Oncology	1	1	2	4
Radiology	1	3	3	7
SAS	2	2	2	6
Total	12	13	14	39

SAIM, small animal internal medicine; SAS, small animal surgery; CI, confidence interval.

### 3.2 Pajama time: all residents

Specialty (*p* < 0.001) was associated with the likelihood of EMR use during PJT. Radiology residents were significantly less likely to use the EMR during PJT compared to all other specialties (all *p* ≤ 0.008). Residency year was associated with the likelihood of EMR use; first year residents were 4.57 times more likely to use the EMR during PJT than residents in year 3 (OR = 4.57, 95% CI: 1.69, 12.36, *p* = 0.002). For those who used the EMR during PJT in a given month, the median time spent across all specialties was 13.57 h/resident/month (95% CI: 0.01, 73.35).

### 3.3 Pajama time by specialty and year

The model estimated medians showed that small animal surgery residents had the highest number of hours spent on the EMR during PJT, with a median of 24.91 h/month (95% CI: 17.72, 35.03), followed by cardiology, 22.31 h/month (95% CI: 13.98, 35.60), neurology, 15.53 h/month (95% CI: 8.31, 29.01), SAIM, 12.40 h/month (95% CI: 8.07, 19.04), anesthesiology, 5.65 h/month (95% CI: 3.03, 10.54), oncology, 5.37 h/month (95% CI:1.59, 18.16), and radiology, 1.33 h/month (95% CI: 0.47, 3.76) PJT. Residents from surgery (*p* < 0.001), cardiology (*p* < 0.001), neurology (*p* = 0.005) and SAIM (*p* = 0.007) all had significantly higher median numbers of hours of PJT compared to radiology residents. Table 2 shows the model-estimated medians and 95% confidence intervals of PJT by specialty. There was no difference in the amount of PJT spent between different years of residency training (*p* = 0.667).

**Table 2:** Model-estimated median EMR use hours during pajama time by specialty.

Specialty	Estimated median	95% CI
Anesthesiology	5.65	3.03–10.54
Cardiology	22.31	13.98–35.60
SAIM	12.40	8.07–19.04
Neurology	15.53	8.31–29.01
Oncology	5.37	1.59–18.16
Radiology	1.33	0.47–3.76
SAS	24.91	17.72–35.03

SAIM, small animal internal medicine; SAS, small animal surgery; CI, confidence interval.

### 3.4 Weekend time: all residents

Specialty (*p* < 0.001) was associated with the likelihood of EMR use during WT. Radiology residents were significantly less likely to use the EMR during WT compared to residents from neurology, SAS, cardiology, SAIM, and oncology (*p* < 0.05). For those who used the EMR during WT in a given month, the median time across all specialties was 23.38 h/resident/month (95% CI: 0.25, 91.60). Residency year was not associated with the likelihood of EMR use during WT (*p* = 0.470).

### 3.5 Weekend time by specialty and by resident year

The model estimated medians showed that neurology residents had the highest number of hours of WT, with a median of 37.44 h/month (95% CI: 26.44, 53.01), followed by cardiology, 36.30 h/month (95% CI: 26.88, 49.03), small animal surgery, 35.76 h/month (95% CI: 26.8, 47.72), SAIM, 20.36 h/month (95% CI: 13.88. 29.86), anesthesiology, 9.94 h/month (95% CI: 5.78, 17.10), oncology, 9.63 h/month (95% CI: 5.29, 17.54), and radiology, 4.87 h/month (95% CI: 3.05, 7.78). Residents from neurology (*p* = 0.003), cardiology (*p* = 0.006), and small animal surgery (*p* < 0.001) had significantly higher EMR hours during WT than anesthesiology, oncology, and radiology residents. SAIM residents had significantly higher median EMR use during WT than radiology residents (*p* < 0.001). Table 3 shows the model estimated medians and 95% confidence intervals of WT by specialty. There was no difference in the amount of WT spent across different years of residency training (*p* = 0.983).

**Table 3:** Model-estimated median EMR use hours during weekend time by specialty.

Specialty	Estimated median	95% CI
Anesthesiology	9.94	5.78–17.10
Cardiology	36.30	26.88–49.03
SAIM	20.36	13.88–29.86
Neurology	37.44	26.44–53.01
Oncology	9.63	5.29–17.54
Radiology	4.87	3.05–7.78
SAS	35.76	26.80–47.72

SAIM, small animal internal medicine; SAS, small animal surgery; CI, confidence interval.

## 4 Discussion

The purpose of our study was to quantify after-hours EMR use among veterinary residents, to compare this across specialties and years of training, and to establish baseline metrics for future studies evaluating documentation efficiency and workflow interventions. To do this, we looked at 2 metrics of describing after-hours work: pajama time (PJT), and weekend time (WT). Our study found that trainees spent a substantial amount of time after-hours on the EMR, and this varied both by individual and by specialty. Across all training programs, for residents who used the EMR during after-hours time in a given month, the median time spent was 13.57 h/month for PJT and 23.38 h/month for WT. This study provides the first objective benchmark for after-hours EMR use among veterinary residents.

While there are no comparable studies in veterinary medicine, there is an ever-growing body of work on EMR use among physicians (1–9, 11, 15, 16, 19–28). These studies have identified similar trends that are consistent with our study, specifically, that physicians spend a substantial amount of time outside of work on the EMR. Rotenstein et al. (20) studied clinicians from a variety of medical and surgical specialties and found that providers had 3.5 h of “work outside of work” for every 8 scheduled patient hours (9). Similarly, Rule et al. (2) studied academic primary care clinicians and found that they averaged 2.9 h of EMR use outside scheduled patient hours and 0.9 h of pajama time per 8 scheduled patient hours. While recent veterinary house officer training guidelines have proposed duty hour limits on veterinary trainees to mirror human guidelines and limit duty hours to no more than 60 h per week averaged over a 4-week period (29), there is limited information on how much time house officers spend on work duties. A recent multi-institutional survey-based study of 303 veterinary interns and residents found that 58% of veterinary interns and residents reported working 11–13 h on a typical weekday, and 32% reported clinical responsibilities 7 days/week (30). This study, which focused on self-reported measures of work hours, did not attempt to quantify the amount of time spent on clinical duty with patients versus time spent outside of patient care hours on the EMR.

In addition to describing the global amount of time spent by residents on the EMR after-hours, we also compared this across different specialties and years of training. Understanding the magnitude of these global job responsibilities is important for attending clinicians and residency program directors, especially given the fact that the majority of after-hours work may occur outside an attending clinician’s direct oversight. For PJT, the top four specialties with the most hours were: SAS, cardiology, neurology, and SAIM. Radiology residents were less likely to use the EMR during PJT time than residents of all other specialties. When they did use the EMR, they had lower median number of hours compared to residents from SAS, cardiology, neurology, and SAIM. Weekend time on the EMR followed a similar pattern. Residents from neurology, cardiology, and SAS had significantly higher EMR use during WT time than anesthesiology, oncology, and radiology residents. There may be several explanations for these findings. Surgery, cardiology, and neurology, by nature of their design, are specialties with a high volume of emergent after-hours procedures and consultations, which likely results in increased time on the EMR. While radiology does incorporate some after-hours emergencies and on-call duties, this specialty focuses on interpretation of imaging studies and consultations, and does not involve primary patient responsibilities, such as the writing of clinical notes and orders, which constitute the bulk of physician time on the EMR (5, 22). In addition, the imaging software to evaluate radiographs and advanced imaging is separate from the EMR, so it is very likely that workload of radiology residents is not fully captured by the EMR metric. In addition to the inherent differences in workflow and EMR usage patterns between specialties, it is also possible that other differences in PJT and WT may have been influenced by the clinic scheduling or caseload. In our study, we defined PJT as before 7:00 a.m. and after 7 p.m. Monday–Friday, and WT was defined as any EMR usage occurring between 12:00 a.m. Saturday and 11:59 p.m. Sunday. While residents at our institution typically work a 5-day workweek (in addition to on-call and weekend responsibilities), it is possible that differences in after-hours EMR time between specialties may also be driven by other factors, such as the number of scheduled weeks on clinic duty, or the number of scheduled weekends on call, which may vary by specialty program. These potential differences in scheduling across specialties may have impacted our findings, and this represents a study limitation. Additionally, the fixed 7:00 a.m. to 7:00 p.m. window used to define PJT may partly reflect inherent shift-time differences across specialties rather than purely after-hours documentation, and radiology’s numbers are likely undercounted since their primary diagnostic imaging software sits outside the core EMR. In order to overcome this limitation, future studies could control for the effects of scheduling by calculating PJT and WT on a per-patient basis or use residency scheduling data to calculate the amount of after-hours EMR time per scheduled 8 h of clinic time (2, 9). It is important to note that our study goal was to capture the holistic resident experience across specialties and generate baseline information regarding the amount of time residents spend on the medical record, irrespective of scheduling. Understanding these general baselines provide a helpful baseline for future studies and interventions.

While we found clear differences between specialties with regards to time spent on the EMR, there was also a substantial amount of variation within specialties. This variability is consistent with previous research, which has identified considerable individual variation in use of the EMR, even among clinicians of the same specialty (8, 16, 19, 25). Anderson et al. described after-hours work on the EMR among family medicine clinicians at a single institution and identified wide variations in time spent on the medical record after-hours among clinicians. This study, which examined 140 residents at a single institution, found that third year residents spent a median of 10.4 h on the EMR per month outside of work, with a range of approximately 1 h to 26 h (26)A separate study examined variability among over 5,000 primary care clinicians and found that individual factors, even more so than health system or practice group, accounted for the largest proportion of variability in EMR use (8) Reasons for this are likely multifactorial and may be related to factors such as gender (7), number of inbox messages received (5), amount of time spent on documentation (5, 8) or individual work preferences (16, 26).

In addition to differences at the specialty level, our study also found that first year residents were more likely to use the EMR during PJT than third year residents. However, among those that did access the EMR during PJT or WT, the median time spent did not differ across different years of training. The reasons for this are likely complex. While first year residents may be less efficient in their use of the EMR and require more time to complete daily tasks required as a clinician, the increased caseload and responsibilities accumulated by the more senior residents as they progress in the program may cancel out any efficiency gains. Studies in physician medicine have yielded mixed results on this (31–33). While residents may become more efficient with EMR use as residency training progress, an increasing caseload with residence may cancel out these gains in efficiency, resulting in a similar amount of after-hours time spent (31).

In addition to the limitations discussed previously regarding the variability in clinical duty schedules between different services, our study includes some additional limitations. Many of the studies evaluating physician EMR use employ vendor-derived EMR metrics (2, 4, 9, 25) to calculate measures such as total time spent on the EMR, PJT, and WT. In contrast, EzyVet, the EMR used at our institution, does not have built-in metrics to automatically capture these data, and these had to be manually calculated. It is possible that a systematic bias in our calculations may have over- under-estimated the time spent on the medical record. While studies in physician literature have used vendor-derived metrics that look at “active” time on EMR as defined by mouse clicks (25) we looked at login and logout time to infer periods of EMR activity. While the EzyVet EMR logs users out after 20 min of inactivity, it is possible that a portion of the time we measured was not truly active work. Other methods to measure work outside of work on the EMR include: self-reporting (22) and direct observation (34), and these represent avenues for future studies. We selected the method used in this study because it was the most straightforward way to retrospectively obtain data from the largest group of individuals (9). Other limitations to the current study include the fact that we did not collect information regarding the gender of participations (7), and the fact that our population was a relatively small size from a single institution, which may impact the generalizability of our results. Furthermore, the relatively small sample sizes within certain specialties and training years limited statistical power and generalizability in this study. Comparisons between specific training years or across specialties should be interpreted with caution. Future prospective studies could collect gender demographic information to look for an association between gender and total EMR time and could also include additional specialties from more than a single institution.

In this study, we found that veterinary residents spent a substantial amount of time after-hours on the electronic medical record, and this varied both by individual and by specialty. While many studies have looked at documentation workload and work outside of work on the EMR (1–11, 16, 19–23, 25–28, 31–34) there are no clear guidelines on after-hours EMR work, or the ideal balance of work outside of hours compared with work within hours. Studies have shown that increased time on the EMR is associated with burnout in physicians (11, 21–23, 27, 28) but this relationship is likely complex and likely multifactorial. A recent survey of clinical faculty from multiple specialties identified that spending more than 90 min per day on the EMR outside of work was associated with a 90% increased risk of burnout (22). Based on these study results, Pecarallo et al. (22) recommended interventions that focused on reducing EMR time and clerical burden to less than an hour or so a day. Some examples of this might include the: use of scribe services, use of a clinical services liaison to assist with client communications and prescriptions and targeted EMR training for clinicians (22). These interventions represent promising avenues for future investigation in the veterinary field. It is important to note that the relationship between time on the EMR and burnout is a complicated one, and other factors, such as perceived usability of the EMR (23, 24) may also play an important role in the relationship between EMR use and burnout. Our current study did not explore the associations between after-hours use of the EMR and burnout and this represents an area of future investigation. As more veterinary colleges employ technologies to assist with documentation, future studies could also investigate the relationship between workflow interventions, including ambient AI scribes and AI-assisted documentation, on after-hours EMR use (20). The methodology presented in this study establishes the first benchmark for after-hours EMR use among veterinary residents and provides a standardized framework for evaluating future interventions. Our study included data from the July 2023–July 2024 training year, before widespread adoption of AI-assisted clinical documentation at our institution.

Future studies currently underway will explore additional factors that impact after-hours time on the EMR, including the use of clinical artificial intelligence tools. As more veterinary hospitals adopt EMRs (8) and clients increasingly communicate with veterinarians via e-mail, reliance on the EMR will likely continue to grow. Faculty members who mentor trainees should be aware of the extent of after-hours work and its potential impact on global workload and educational experiences.

## Data Availability

The datasets presented in this article are not readily available because the datasets are not publicly available because they contain institutional electronic medical record metadata. Requests for access will be considered by the corresponding author in accordance with institutional data governance and participant confidentiality requirements. Requests to access the datasets should be directed to kemccool@ncsu.edu.
